# K-core decomposition of a protein domain co-occurrence network reveals lower cancer mutation rates for interior cores

**DOI:** 10.1186/s13336-015-0016-6

**Published:** 2015-03-03

**Authors:** Arnold I Emerson, Simeon Andrews, Ikhlak Ahmed, Thasni KA Azis, Joel A Malek

**Affiliations:** Department of Genetic Medicine, Weill Cornell Medical College, New York, NY USA; Genomic Core, Weill Cornell Medical College in Qatar, Qatar Foundation, Doha, 24144 Qatar

**Keywords:** Domain co-occurrence network, K-core decomposition, Somatic mutations, Cancer, Cancer mutations, TCGA

## Abstract

**Background:**

Network biology currently focuses primarily on metabolic pathways, gene regulatory, and protein-protein interaction networks. While these approaches have yielded critical information, alternative methods to network analysis will offer new perspectives on biological information. A little explored area is the interactions between domains that can be captured using domain co-occurrence networks (DCN). A DCN can be used to study the function and interaction of proteins by representing protein domains and their co-existence in genes and by mapping cancer mutations to the individual protein domains to identify signals.

**Results:**

The domain co-occurrence network was constructed for the human proteome based on PFAM domains in proteins. Highly connected domains in the central cores were identified using the k-core decomposition technique. Here we show that these domains were found to be more evolutionarily conserved than the peripheral domains. The somatic mutations for ovarian, breast and prostate cancer diseases were obtained from the TCGA database. We mapped the somatic mutations to the individual protein domains and the local false discovery rate was used to identify significantly mutated domains in each cancer type. Significantly mutated domains were found to be enriched in cancer disease pathways. However, we found that the inner cores of the DCN did not contain any of the significantly mutated domains. We observed that the inner core protein domains are highly conserved and these domains co-exist in large numbers with other protein domains.

**Conclusion:**

Mutations and domain co-occurrence networks provide a framework for understanding hierarchal designs in protein function from a network perspective. This study provides evidence that a majority of protein domains in the inner core of the DCN have a lower mutation frequency and that protein domains present in the peripheral regions of the k-core contribute more heavily to the disease. These findings may contribute further to drug development.

**Electronic supplementary material:**

The online version of this article (doi:10.1186/s13336-015-0016-6) contains supplementary material, which is available to authorized users.

## Background

Domains are distinct functional or structural units in a protein. Most domains correspond to tertiary structure elements, and are able to fold independently. All protein domains exhibit evolutionary conservation and many either perform specific functions or contribute in a specific way to the structure of their proteins. Domains may exist in a variety of biological contexts, wherein similar domains can be found in proteins with different functions. Many proteins are composed of one or more domains that can fold independently into a stable core structure [1-3].

Many complex systems have been analyzed as networks by representing the system as nodes and interactions between them as edges. Studies on complex networks including the network of co-authorships, sexual contacts and the world-wide-web (WWW) reveal that their structure and growth is governed by a set of generic organizing principles [4,5]. Network biology is emerging as a new field in biology due to the increasing availability of genome-scale data sets of molecular interactions. These data are a result of new high-throughput technologies yielding information on protein interactions, regulatory networks and the metabolome. Biological systems like gene interaction networks, protein and metabolite networks have been found to exhibit a scale-free property [6-13].

The development of high-throughput, whole-exome/genome DNA sequencing has made it possible to evaluate normal and tumor tissue samples in a single study. These studies have revealed the connection between somatic mutations and cancer susceptibility, initiation and development [14]. A central goal of cancer genome analysis is the identification of cancer genes that, by definition, carry driver mutations. A key challenge will therefore be to distinguish driver from passenger mutations. Most studies thus far have attempted to identify driver mutations using gene-centric approaches [15-20]. Unfortunately, this method is limited to a small subset of genes and also leads to mischaracterized mutations [21]. The gene-based approach usually fails to reflect the position of mutation or the functional context the position of mutation provides in protein level. But a protein domain network enables the identification of mutations that are rare at the gene level, but that occur frequently within the specified domain. These highly mutated domains potentially reveal disruptions of protein function necessary for cancer development.

Several studies have been conducted on protein domain co-occurrence networks (DCN). These studies represent domains as nodes and their co-occurrence in a protein are denoted as edges. The networks have also been shown to possess a scale-free property [22-24]. Increasing complexity of the organisms were observed from bacteria to eukaryotes due to the links involved in the cell-cell interaction domains, signal transduction and cell differentiation domains. Studies on DCN have examined the network property [22], evolutionary traces among the species [25], architectural design of protein domain networks [26] and mapping somatic mutations to protein domains in colon cancer [27]. More recently, a disease-drug-phenotype matrix was also analyzed using protein domain networks [28]. However, each of these studies have focused either on domain co-occurrence networks or on a specific feature of the DCN and therefore, do not provide a generalized view of mutations in the domain co-occurrence network. In this study, we investigated the protein domain co-occurrence network in the context of various cancers and their mutations. We specifically focused on the highly connected protein domains of the DCN core by using k-core decomposition techniques.

The definition of k-core was first introduced by Seidman [29] to characterize the cohesive regions of graphs. Batagelj et al., developed an efficient algorithm to find the k-core decomposition of a graph [30]. K-core technique has been used in many areas including the alternative method for community detection algorithm [31] and for the identification of dense components in most of the complex networks [32-34].

K-core decomposition is a network analysis approach that helps in understanding interesting structural properties that are not otherwise captured by many other network topological parameters. The basic principle behind the k-core is decomposition to identify particular subsets of the network called k-cores. Each k-core is obtained by a recursive pruning method [29,35,36]. This decomposition method allows the study of the hierarchical properties of large complex networks by focusing on the network centrality and connectedness properties of nodes. The central cores of this analysis have more strongly connected vertices with large number of possible distinct paths between them. This helps in obtaining robust routing properties.

## Materials and methods

### Construction of protein domain co-occurrence networks (DCN)

The DCN for *Homo sapiens* was constructed using the Ensembl database (version 72) that provides a comprehensive source of stable automatic annotation of individual genomes [37].

**Figure 1:**
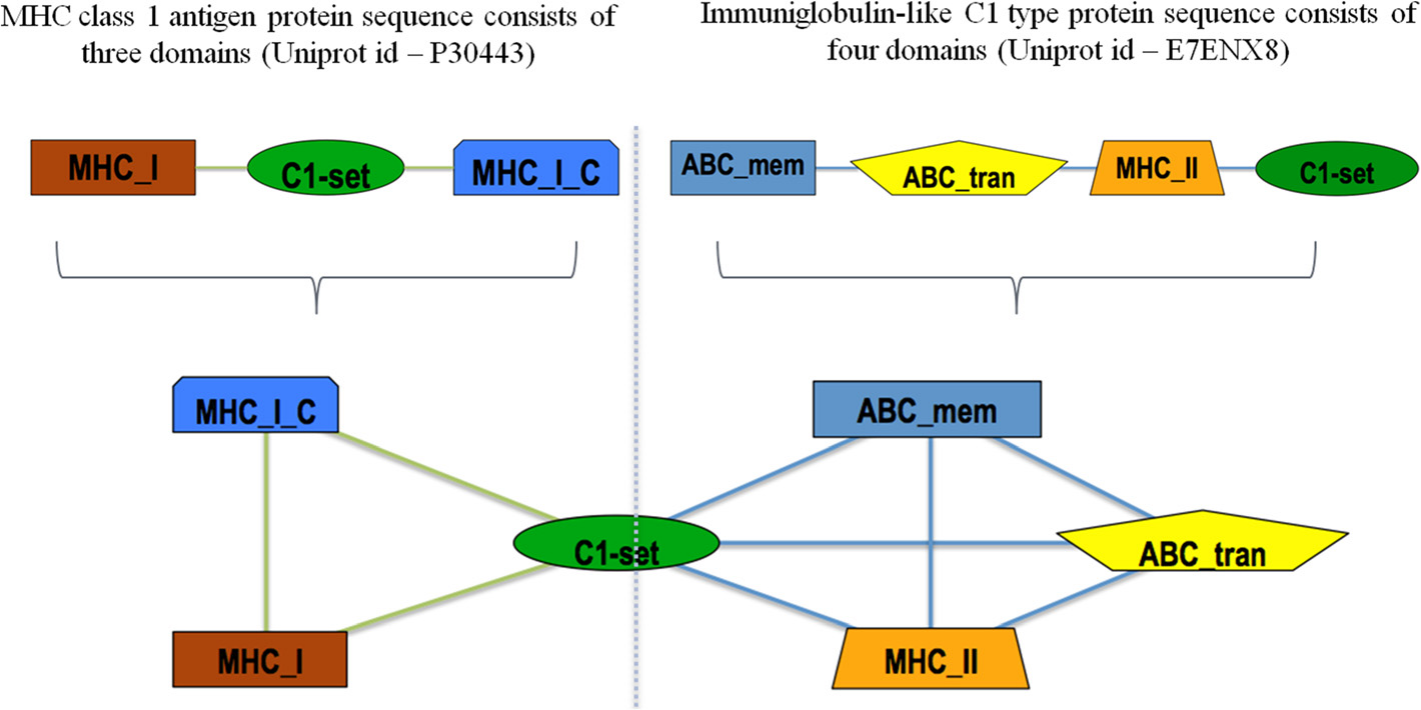
**A small DCN consisting of two** ***H. sapiens*** **proteins.** The two proteins share the same domain C1-set. An edge is drawn between domains co-occurring in the same protein and a fully connected sub-graph (clique) in the DCN corresponds to a protein.

**Figure 2:**
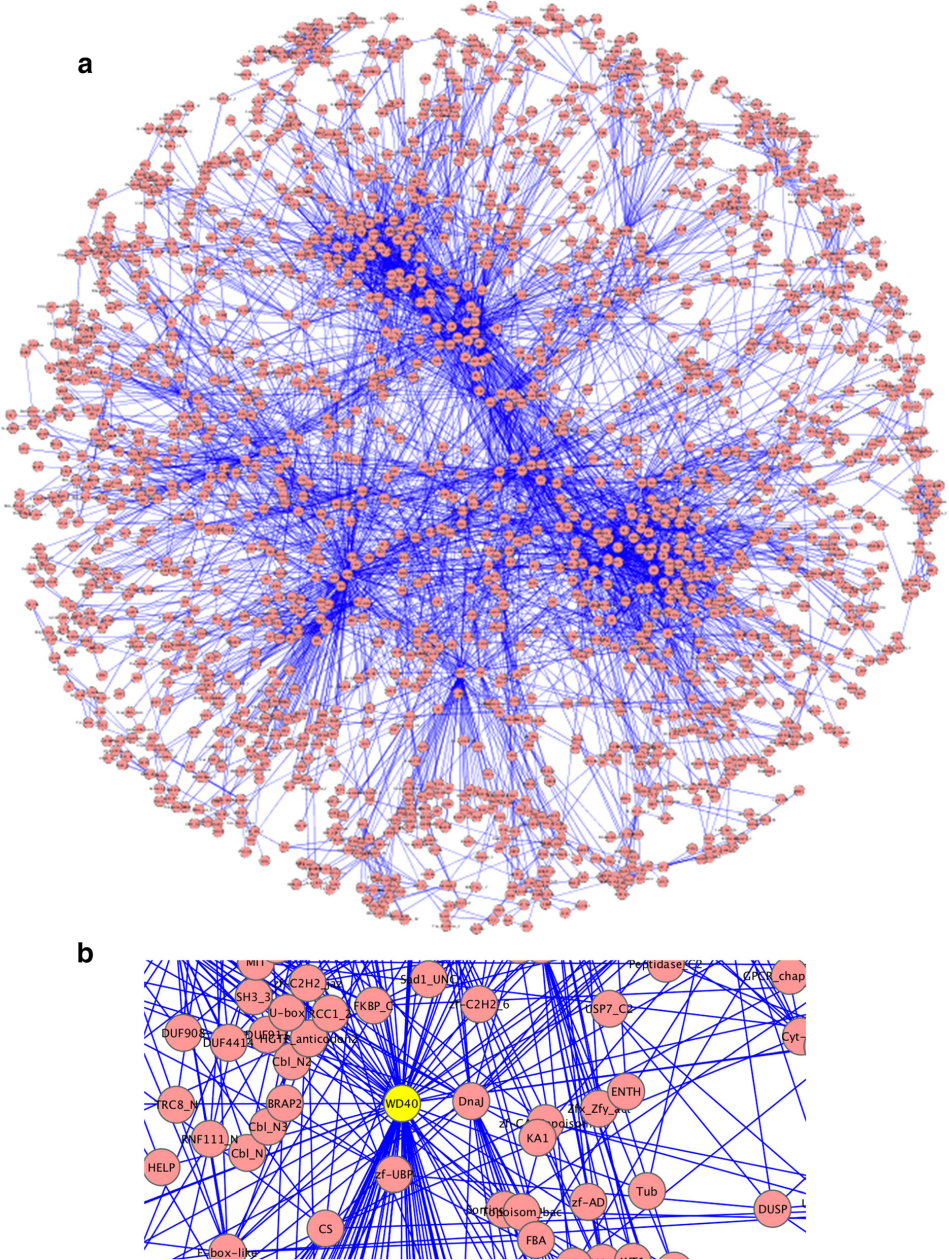
**Domain co-occurrence network of** ***H. sapiens.*** **(a)** The largest DCN sub-graph or the main graph of *H. -sapiens*, which consists of 1929 nodes and 5171 edges. **(b)** The enlarged partial view of *H. sapiens* main DCN, in which the domain WD40 (beta-transducin repeat) is represented as a hub.

To ensure correct coordinates in our version domains present in any protein sequence of the human proteome were determined using the program PfamScan [38]. Domain hits with an e-value < =0.01 were considered for constructing a DCN. Each domain was represented as a node and if every two domains co-exist in one protein then they were connected by edges as shown in Figure 1 [25]. Figure 2 illustrates the largest sub-graph for *H. sapiens* consisting of 1929 nodes and 5171 edges as visualized by Cytoscape [39].

### K-core decomposition of DCN

To identify the core-periphery organization of the domain co-occurrence network, we subjected it to core decomposition. The cores of different orders of a network can be obtained by iteratively removing all nodes which have less than *k* connections with other protein domains (*k* = 1, 2, …). This is done by first identifying all nodes whose degree (i.e., number of connections) is less than *k*. After removing these, the network is re-analyzed to determine if the removal of these nodes has resulted in other nodes (which originally had degree > *k*) having now less than *k* connections. If such nodes are identified, then they are removed, and the process is continued, until no more nodes can be removed. The resulting sub-network is called the *k*-core of the network.

### Randomization of k-core

To determine the statistical significance of the properties calculated for members of an empirically determined *k*-core, we compared them with the mean and variance of the corresponding values obtained for a randomized ensemble. Each randomized *k*-core in the ensemble is obtained by random selection without replacement of N_k_ domains from the DCN, where N_k_ is the size of the empirically determined *k*-core. The randomized ensemble for every DCN considered was generated by constructing 100 such randomized *k*-cores.

### Evolutionary conservation of protein domains

The evolutionary conserved protein domains were identified using the database PANDIT*plus* [40]*.* It consists of a database of PFAM alignment phylogenetic trees for known protein domains and their families. This database was constructed using a relational database which comprises of information regarding the functional categories, metabolic pathways, protein–protein interactions, disease associations, gene expressions, three-dimensional structures, as well as estimates from an evolutionary analyses of selective pressures.

### Cancer mutation dataset

Somatic mutation data for ovarian, breast and prostate cancer were obtained from TCGA data portal (http://tcga-data.nci.nih.gov/tcga/) using mutation files from the hgsc.bcm.edu_COAD.IlluminaGA_DNASeq.1 and hgsc.bcm.edu_COAD.SOLiD_DNASeq.1 directories downloaded on March 30^th^, 2013. The silent and RNA mutations were filtered out from the data set as they were assumed unlikely to affect the cancer development. Somatic mutation counts for ovarian cancer were found to be 20,878. For breast and prostate cancer the values were found to be 35,558 and 23,349.

### Mapping cancer SNPs to individual protein domains

Before mapping mutations to the individual protein domains, the protein domain positions need to be converted into their chromosome positions. Mutations obtained from TCGA data portal were reported with genomic locations while predicted PFAM domains documented in peptide coordinates. A Perl program was written using the ensemble Perl API module for converting the protein domain positions into chromosome positions. The Pfam domains from *Homo sapiens* were successfully mapped with the chromosome positions as shown in the Additional file 1: Table S1. For ovarian cancer sample set, almost one third of the mutations (30%) occurred inside annotated protein domain regions. Similarly 47.5% and 49.3% of all mutations in breast and prostate cancer sample sets were observed to have occurred inside the protein domain space (Table 1).

**Table 1:** **Percentage of mapped mutations in three forms of cancer**

**S. No**	**Cancer type**	**No. of Mutations**	**No. of mapped mutations**	**Percentage**
1	Ovarian	20,878	5,842	30%
2	Breast	35,558	16,887	47.5%
3	Prostate	23,349	11,502	49.3%

### Procedure for normalizing the domain mutation frequency

To determine the domains that are frequently mutated in the human genome, we first obtained the count of mutations that fell within each domain. Since larger domains are generally expected to accumulate more mutations than the shorter domains, we normalized the domain mutation counts with domain length. This was done by dividing domain mutations counts by the cumulative length of the domain in the genome. That is, the summed length of all occurrences of the domain in the genome was used as total length. The normalized score for all the three cancer types are shown in Additional file 2: Table S2.

### Calculation of significantly mutated domains

Domain mutation counts were normalized with the cumulative length of the domain in the genome. We then obtained the relative frequencies from the normalized values and these frequencies were used as success probability (p). This probability (p) was normalized using the signal to noise ratio of the Bernoulli distribution, which resulted in a normalized score, z and is given by

$$ Z=p/ sqrt\left(p\left(1-p\right)\right) $$

The **“locfdr”** package [41] from R was used to estimate the null distribution and these statistics were used to identify domains with a local false discovery rate < 0.1 [42]. The local false discovery rate values for each domain in all the three cancer types are shown in Additional file 3: Table S3a (sheet1), Table S3b (sheet2) and Table S3c (sheet3).

## Results

### Domains in the inner cores are more conserved than those at the periphery

The domain co-occurrence network of *Homo sapiens* was constructed and its statistical properties were determined. From the degree distribution plot (Additional file 4: Figure S1a), the DCN was found to have scale-free behavior. Additional file 4: Figure S1b shows the shortest path length distribution exhibiting a small-world phenomenon. The average clustering coefficient distributions and the node degrees are found to have an inherent hierarchical modularity (Additional file 4: Figure S1c). We applied the k-core decomposition algorithm to the *Homo sapiens* DCN [29]. The cores were found to have 10 nested k-cores, where k values ranged from 1 to 10. The property of k-core decomposition is that as the core increases the number of nodes in each core decreases. This property was observed in the *Homo sapiens* DCN.

**Figure 3:**
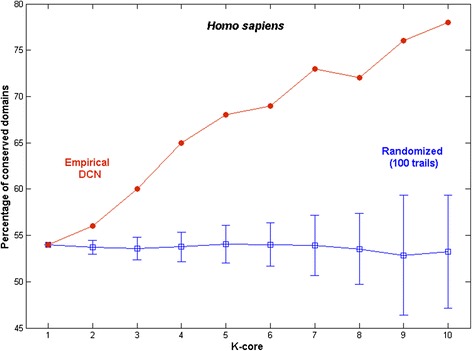
**The percentage of conserved domains within each core order.**

To differentiate the protein domains in each core, we first identified the conserved domains in each core using PANDIT server. To verify whether the frequency of conserved domains in the inner cores is statistically significant, the empirical values were compared against the randomized cores. The percentage of conserved domains increased with increasing core order in contrast to the random cores that did not show significant deviations with the core order (Figure 3). The deviation of the empirical data with core order is much greater than the error bars obtained from the random ensemble. This suggests that the highly conserved nature of the inner core domains is significant in the empirical DCN.

### Domains in inner cores have fewer mutations than those at the periphery

Cancer mutations obtained from TCGA data portal were mapped to the individual protein domains. The normalized mutation score for each protein domain was also calculated (for details see Methods). To study the mutations in domain co-occurrence network, the normalized mutation scores were assigned to all the nodes (i.e., protein domain) in the network. In order to understand the nature of mutation in the *Homo sapiens* DCN we subjected it to core decomposition. We found that the normalized mutations per domain in each core gradually decreased with the core order. This observation occurred for all the three cancer types. To verify whether the findings are statistically significant, we compared the empirical DCN with its random counterparts. The pattern observed in the *Homo sapiens* DCN was not found in 100 random DCNs (i.e. p < 0.01).

**Figure 4:**
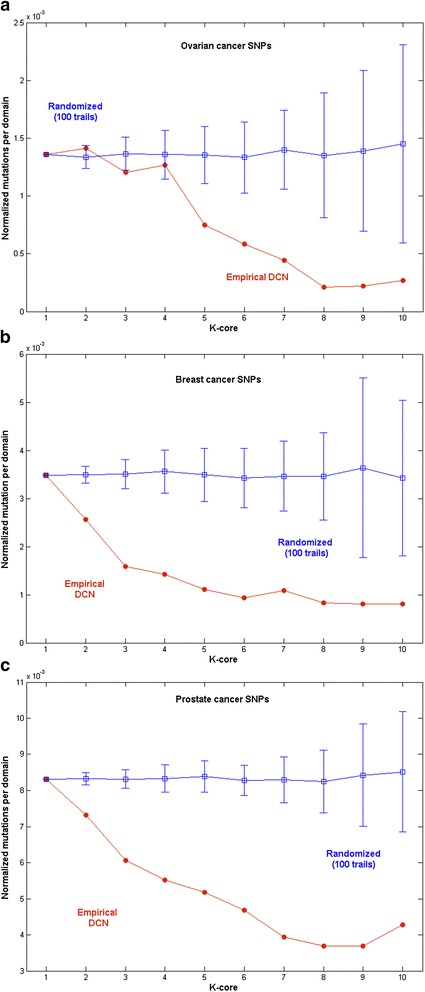
**Variation of normalized mutation values with the core order a) Ovarian cancer b) Breast cancer and c) Prostate cancer.**

As shown in Figure 4, the number of normalized mutations per domain corresponding to the random cores does not show any significant deviation with core order, unlike the case for the empirical DCN. The deviation of the empirical data with core order is much less than the error bars obtained from the random model. This suggests that the less mutated nature of the inner core domains is significant. A similar study shows that the domains in the inner cores of *S.cerevisiae*, *C.elegans, D.melanogaster, M.musculus and H. sapiens* have been preserved during evolution. This high conservation of inner core domains across species development may explain why they are also less mutated in comparison to the peripheral protein domains in cancer [25].

From the randomized simulations, we observed that the inner core domains had lesser rates of mutation compared to the peripheral cores. To verify if, the inner core significantly differed from the other cores (we wanted to investigate the extent to which this aspect was true and to also identify outliers), every domain’s normalized mutation rates were plotted against the k-core values for the three types of cancers as shown in Additional file 5: Figure S2 (2a-ovarian cancer, 2b-breast cancer & 2c-prostate cancer). Results suggested that the normalized mutation rates gradually declined with the core order and the correlation values (R^2^) between them were also found to be positive. Interestingly, the outlier of the inner core is found to be more significant as it comprises of lower mutation rates.

### Identification of significant mutated domains

**Figure 5:**
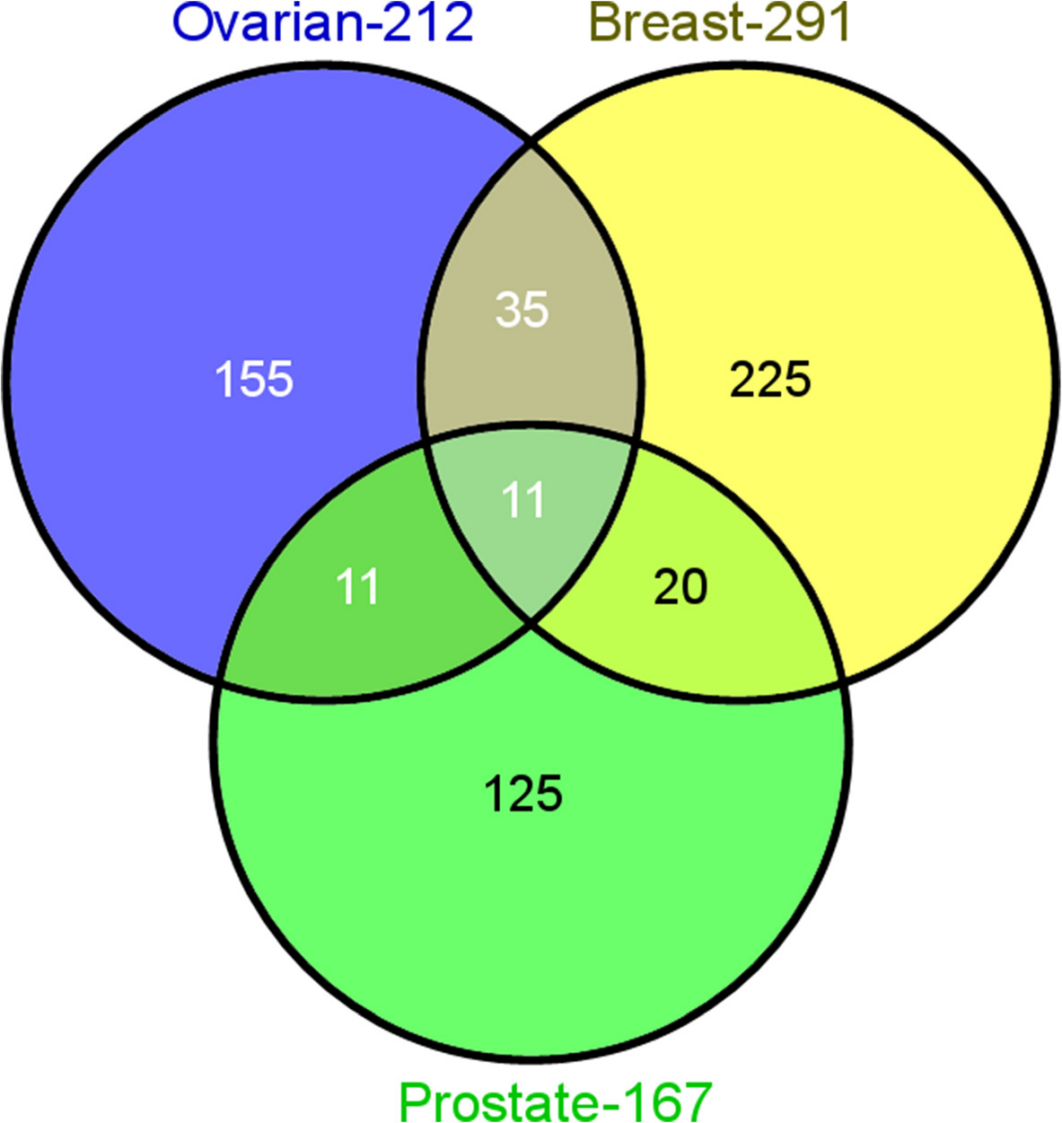
**Overlap between significantly mutated domains of ovarian, breast and prostate cancer.**

Significantly mutated domains in all of the three cancer types were identified using the local false discovery rate. On comparing the significantly mutated domains among the cancer types, we found several domains common between ovarian, breast and prostate cancer (Figure 5). The significantly different domains in all three cancers are tabulated in the Additional file 6: Table S4. Interestingly, we found that in all of the three cancer sets the P53 domain scores the highest number of mutations. Among all the cancer domains 11 pfam domains were commonly mutated (Table 2). To determine the functions overrepresented in our sets of significant mutated domains, we obtained the annotations for all the domains using DAVID [43,44]. A list of KEGG pathways and gene annotation terms from the enrichment analysis of significant domains for ovarian, breast and prostate cancer can be found in the Additional file 7: Table S5. A subsequent enrichment analysis of KEGG pathways annotated for significant PFAM domains in prostate cancer revealed pathways related to Toll-like receptor signaling, small cell lung cancer, complement and coagulation cascades, etc. A similar analysis of GO terms annotated for prostate cancer revealed an overrepresentation of GO terms related to death, development process, and cellular component organization among others. The complete set of KEGG pathway and GO term annotation for ovarian and breast cancer is tabulated in Additional file 7: Table S5a, b.

**Table 2:** **Significantly mutated domains found in all three cancers studied**

**S. No**	**Pfam id**	**Pfam domains**
1	PF00870	P53
2	PF12129	Phtf-FEM1B_bdg
3	PF01192	RNA_pol_Rpb6
4	PF02020	W2
5	PF09801	SYS1
6	PF07941	K_channel_TID
7	PF00594	Gla
8	PF13096	CENP-P
9	PF01250	Ribosomal_S6
10	PF11629	Mst1_SARAH
11	PF05111	Amelin

**Figure 6:**
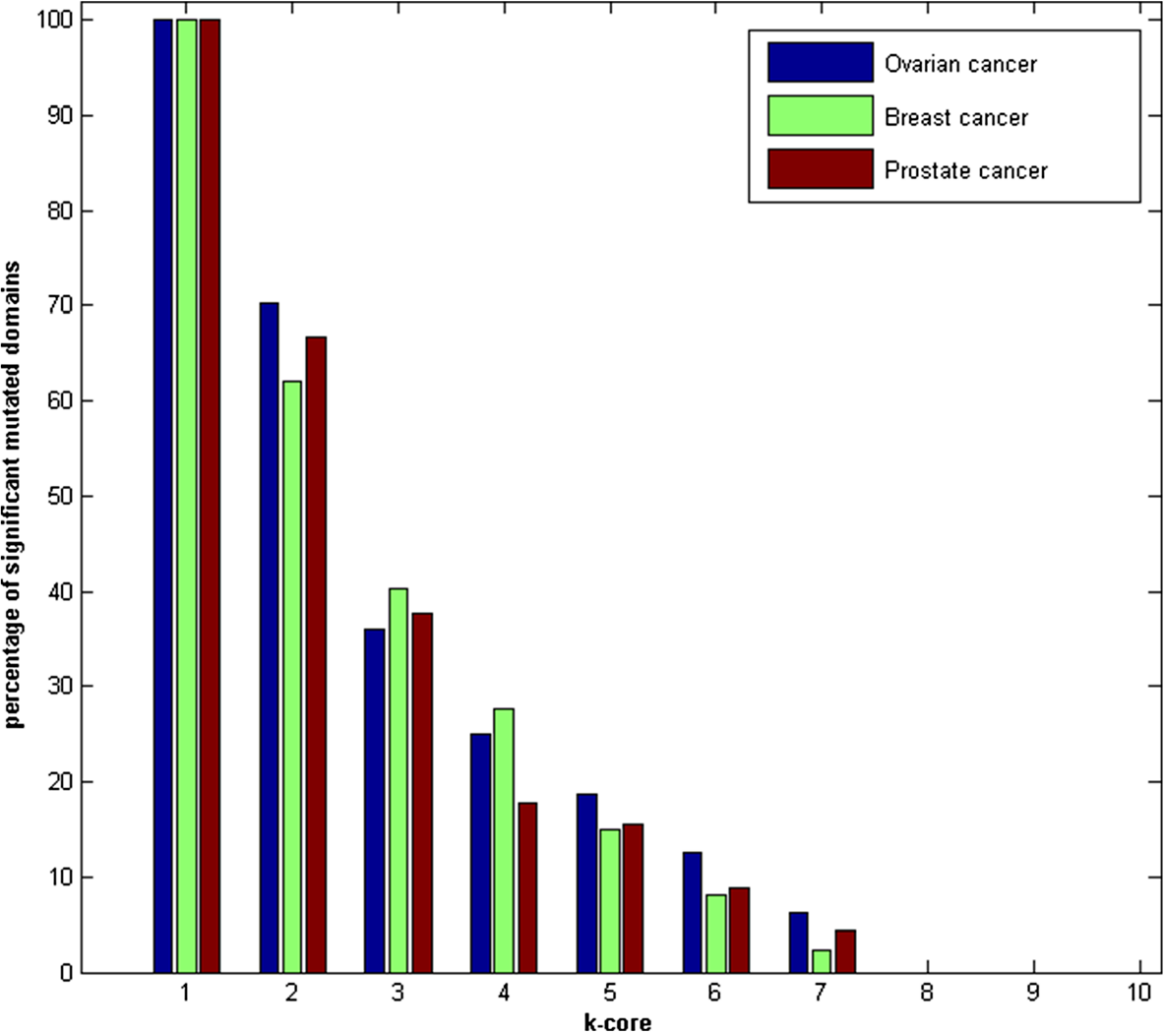
**The percentage of significantly mutated domains in k-core decomposition.**

As discussed in the previous section, core domains were found to be less mutated in comparison to the peripheral domains. We also investigated the presence of significantly mutated domains in each core of the domain co-occurrence network. We calculated the percentages of significant domains in each core of the DCN as shown in Figure 6. Interestingly, we found that inner cores 8, 9 and 10 did not contain any significantly mutated domains. This indicates clearly that the inner core domains have been highly conserved through evolution and also less mutated in cancer. From the study done by Benjamin A. et. al., it was found that highly connected nodes in the domain interaction network had domains which were conserved and also involved in important biological roles within a cell [45].

## Discussion

On analyzing genomes studies have shown that more than 70% of eukaryotic proteins comprised of multiple domains. Domain-domain interactions are now becoming an upcoming trend of interest across numerous studies [46-51]. Studies on protein-protein and domain-domain interaction networks using graph models have revealed that domain levels are the most important aspects of evolutionary selection. In addition to this, protein structural domains seem to have been the most distinct and significant biological entities for interaction, function and evolution [47]. Modeling of domain interaction networks have identified that domains are often involved in the propagation of signal transduction and helps determine the recognition specificity of each domain family member. This becomes an essential step toward a functional description of the global interactome [48].

By constructing and analyzing domain co-occurrence networks we gain new and fundamental insights into the qualitative arrangement and evolutionary utilization of the proteome. Domain databases like Pfam and Interdom provide comprehensive domain information but mapping cancer SNPs to the individual domains may help identify cancer targeted protein domains rather than just the proteins. Domains with high relative rates of mutation in three hormonal cancer types were identified along with their common domains. Recent studies from Liu et al, 2014 revealed that the PDZ and LIM protein domain promotes breast cancer cell migration, invasion and metastasis [52]. These two Pfam domains were also listed among the significantly mutated domains of the breast cancer.

**Figure 7:**
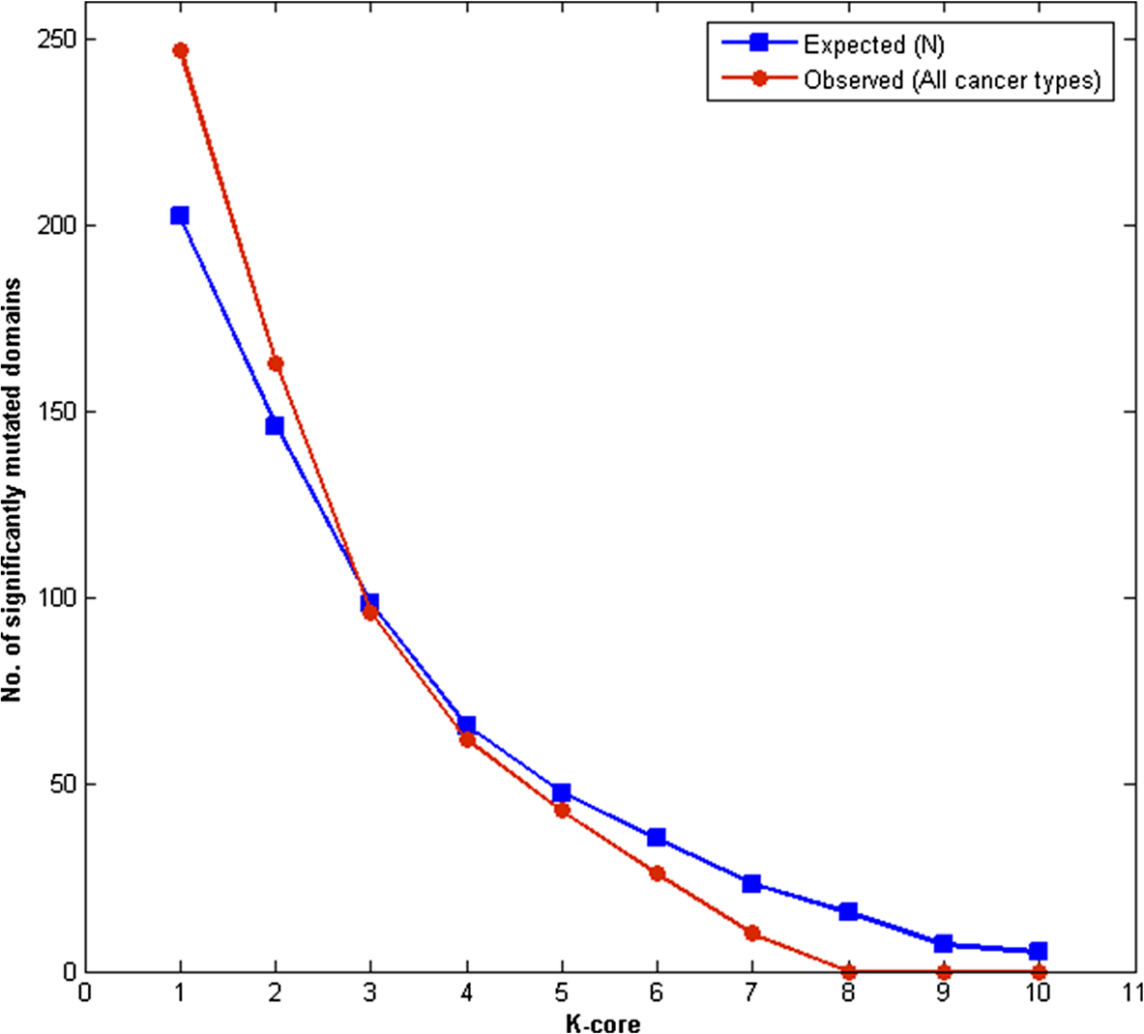
**The expected versus observed significantly mutated domains in the core order.**

Chi-squared goodness of fit test was employed to validate whether the observed and expected mutations are statistically significant. The expected mutations were calculated from the *Homo sapiens* DCN and the observed mutations included all the three cancer SNPs. The expected and observed mutations in different k-cores were calculated and plotted as shown in Figure 7. The observed mutations (red curve) were found to be lower than the expected mutations (blue curve) as the core order increased. This clearly suggests that the protein domain in the innermost core is less likely to get mutated as it was connected to many other protein domains and also corresponding to the set of domains with highest coreness. The p-value was found to be less than 0.05 suggesting that the observed mutation counts are not sampled from populations with the expected frequencies.

## Conclusion

Analyzing mutations in domain co-occurrence network helps in identifying crucial protein domains that aid in the progression of cancer disease. Highly connected protein domains are found to be evolutionarily conserved in the domain co-occurrence network. This implies that protein domains in the inner core are more conserved than the domains in the peripheral region. Significantly mutated protein domains which were identified further contributed to determining the disease target protein domains in all the three cancer diseases. Comparing the mutational landscape of somatic mutations in the protein domain co-occurrence network with the random counterparts, our findings revealed that there is a statistically significant difference between them.

The functional annotations obtained for all the significantly mutated domains were seen to be involved in all the three cancer diseases. Polymorphisms in inflammation-related genes, including those in the Toll-like receptor (TLR) signaling pathway, are hypothesized to be involved in prostate carcinogenesis [53]. This Toll-like receptor signaling pathway was enriched as one of the top ranked KEGG pathway in our results. Similarly, the ribosome pathway is also enriched as one of the top ranked KEGG pathway. This ribosome pathway are activated in aggressive human breast cancer cells [54] and comparison with other pathways showed that the ribosome pathway genes were up-regulated in ZR-75-1 (Human breast carcinoma cell line) [55].

A recent study done by Pasta A et al [56], showed that overexpressed genes in cancer stem cells (CSC) from patients with epithelial ovarian cancer are associated with glucose uptake, oxidative phosphorylation (OXPHOS) and fatty acid beta-oxidation. These overexpressed genes are consistent with a metabolic profile dominated by OXPHOS pathway [56]. In our results, ‘Complement and coagulation cascades’ was the most frequently perturbed pathway, as it was dysregulated in the ovarian cancer [57] and these two pathways are also found to be enriched in the significantly mutated domains. This clearly suggests that the statistically significant domains occur more commonly in cancer diseases. Further studies are however recommended to investigate the functional and structural constraints for the protein domain that evolves to be an inner core rather than outer core domain of the DCN.

## Additional files

Additional file 1: Table S1. Chromosome start and end positions for each predicted domain from homo sapiens proteome. Additional file 2: Table S2a (sheet1). Mutation count and their normalized score for each domain in Ovarian cancer. **Table S2b (sheet2).** Mutation count and their normalized score for each domain in breast cancer. **Table S2c (sheet3).** Mutation count and their normalized score for each domain in prostate cancer. Additional file 3: Table S3a (sheet1). Local false discovery rate values for each domain in ovarian cancer. **Table S3b (sheet2).** Local false discovery rate values for each domain in breast cancer. **Table S3c (sheet3).** Local false discovery rate values for each domain in prostate cancer. Additional file 4: Figure S1a. Degree distribution plot for Homo sapien’s DCN. **Figure S1b.** Shortest path length distribution plot for Homo sapien’s DCN. **Figure S1c.** Average clustering co-efficient distribution plot for Homo sapien’s DCN. Additional file 5: Figure S2a. Correlation between each domain’s normalized mutation rate and k-core values in ovarian cancer. **Figure S2b.** Correlation between each domain’s normalized mutation rate and k-core values in breast cancer. **Figure S2c.** Correlation between each domain’s normalized mutation rate and k-core values in prostate cancer. Additional file 6: Table S4a (sheet1). Significantly mutated domains in ovarian cancer using local false discovery rate values. **Table S4b (sheet2).** Significantly mutated domains in breast cancer using local false discovery rate values. **Table S4c (sheet3).** Significantly mutated domains in prostate cancer using local false discovery rate values. Additional file 7:
**Table S5a (sheet1).** KEGG pathways and gene ontology annotations enriched in significantly mutated domains for the ovarian cancer using DVAID tool. **Table S5b (sheet2).** KEGG pathways and gene ontology annotations enriched in significantly mutated domains for the breast cancer using DVAID tool. **Table S5c (sheet3).** KEGG pathways and gene ontology annotations enriched in significantly mutated domains for the prostate cancer using DVAID tool.
