# Supplementary figures and images for: K-core decomposition of a protein domain co-occurrence network reveals lower cancer mutation rates for interior cores
Source: J Clin Bioinforma. 2015 Mar 3;5:1. doi: 10.1186/s13336-015-0016-6 (PMC4357223; doi:10.1186/s13336-015-0016-6)

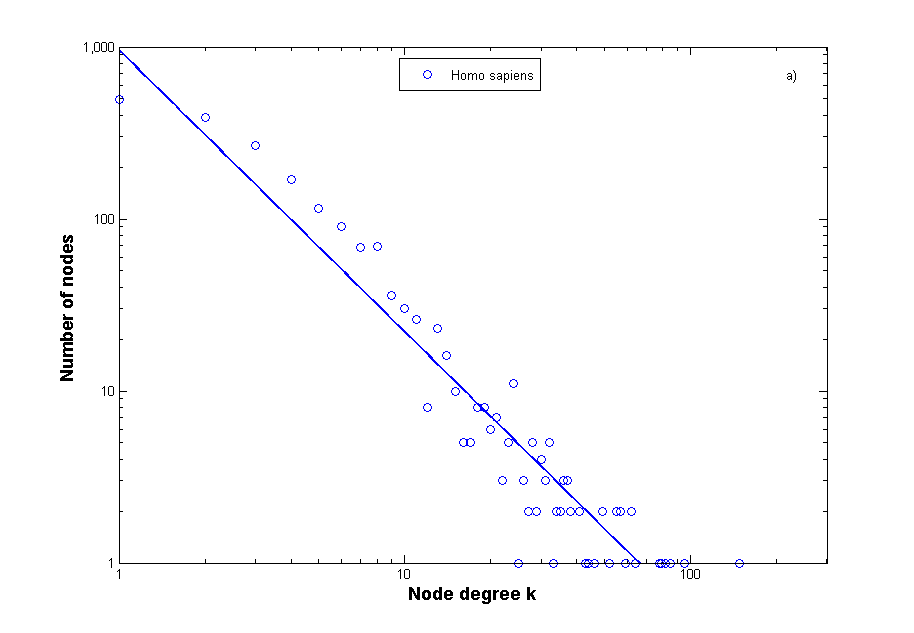

Supplement: Additional file 4: Figure S1a. — Degree distribution plot for Homo sapien’s DCN. Figure S1b. Shortest path length distribution plot for Homo sapien’s DCN. Figure S1c. Average clustering co-efficient distribution plot for Homo sapien’s DCN. [file 13336_2015_16_MOESM4_ESM.zip › add4/9017134321501816_add1a.tiff]

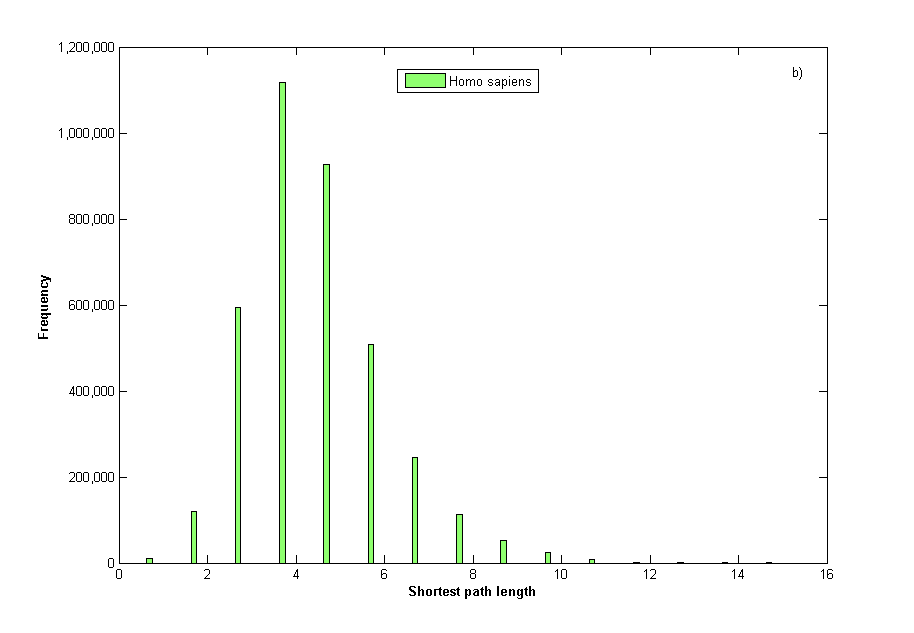

Supplement: Additional file 4: Figure S1a. — Degree distribution plot for Homo sapien’s DCN. Figure S1b. Shortest path length distribution plot for Homo sapien’s DCN. Figure S1c. Average clustering co-efficient distribution plot for Homo sapien’s DCN. [file 13336_2015_16_MOESM4_ESM.zip › add4/9017134321501816_add1b.tiff]

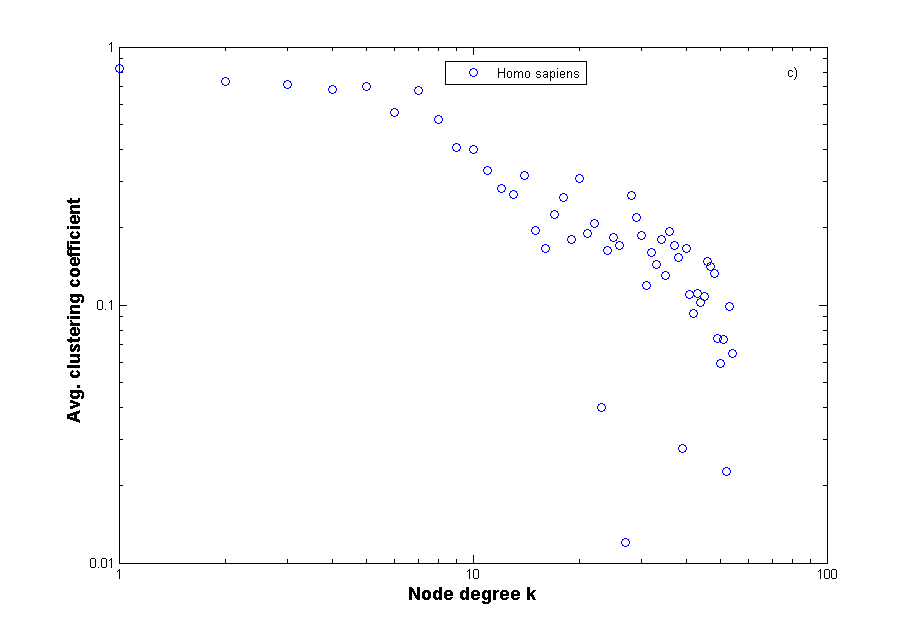

Supplement: Additional file 4: Figure S1a. — Degree distribution plot for Homo sapien’s DCN. Figure S1b. Shortest path length distribution plot for Homo sapien’s DCN. Figure S1c. Average clustering co-efficient distribution plot for Homo sapien’s DCN. [file 13336_2015_16_MOESM4_ESM.zip › add4/9017134321501816_add1c.tiff]

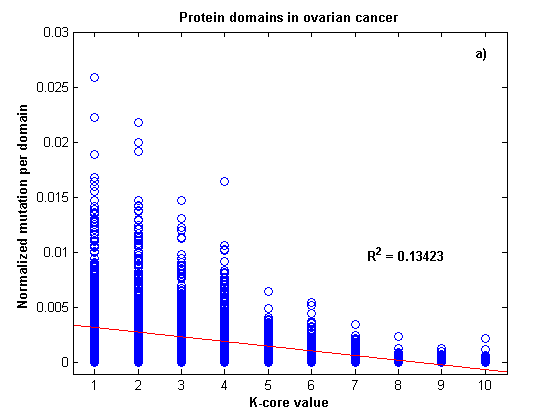

Supplement: Additional file 5: Figure S2a. — Correlation between each domain’s normalized mutation rate and k-core values in ovarian cancer. Figure S2b. Correlation between each domain’s normalized mutation rate and k-core values in breast cancer. Figure S2c. Correlation between each domain’s normalized mutation rate and k-core values in prostate cancer. [file 13336_2015_16_MOESM5_ESM.zip › add5/9017134321501816_add2a.tiff]

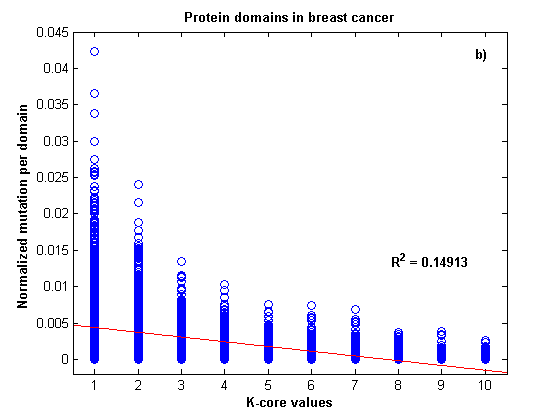

Supplement: Additional file 5: Figure S2a. — Correlation between each domain’s normalized mutation rate and k-core values in ovarian cancer. Figure S2b. Correlation between each domain’s normalized mutation rate and k-core values in breast cancer. Figure S2c. Correlation between each domain’s normalized mutation rate and k-core values in prostate cancer. [file 13336_2015_16_MOESM5_ESM.zip › add5/9017134321501816_add2b.tiff]

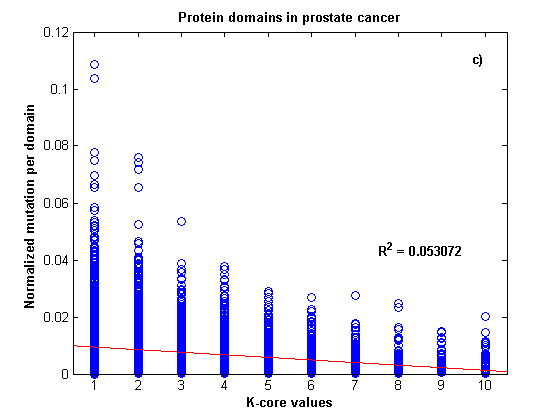

Supplement: Additional file 5: Figure S2a. — Correlation between each domain’s normalized mutation rate and k-core values in ovarian cancer. Figure S2b. Correlation between each domain’s normalized mutation rate and k-core values in breast cancer. Figure S2c. Correlation between each domain’s normalized mutation rate and k-core values in prostate cancer. [file 13336_2015_16_MOESM5_ESM.zip › add5/9017134321501816_add2c.tiff]
